# Identification of four hub genes in venous thromboembolism via weighted gene coexpression network analysis

**DOI:** 10.1186/s12872-021-02409-4

**Published:** 2021-12-03

**Authors:** Guoju Fan, Zhihai Jin, Kaiqiang Wang, Huitang Yang, Jun Wang, Yankui Li, Bo Chen, Hongwei Zhang

**Affiliations:** 1 Department of Vascular Surgery, The Second Hospital of Tianjin Medical University, No. 23, Pingjiang Road, Hexi District, Tianjin, 300211 China; 2 Department of Orthopedics, Handan First Hospital, Handan, China

**Keywords:** Venous thromboembolism, Hub genes, Prognosis, Prediction, Biomarker

## Abstract

**Background:**

The pathogenic mechanisms of venous thromboembolism (VT) remain to be defined. This study aimed to identify differentially expressed genes (DEGs) that could serve as potential therapeutic targets for VT.

**Methods:**

Two human datasets (GSE19151 and GSE48000) were analyzed by the robust rank aggregation method. Gene ontology and Kyoto encyclopedia of genes and genomes pathway enrichment analyses were conducted for the DEGs. To explore potential correlations between gene sets and clinical features and to identify hub genes, we utilized weighted gene coexpression network analysis (WGCNA) to build gene coexpression networks incorporating the DEGs. Then, the levels of the hub genes were analyzed in the GSE datasets. Based on the expression of the hub genes, the possible pathways were explored by gene set enrichment analysis and gene set variation analysis. Finally, the diagnostic value of the hub genes was assessed by receiver operating characteristic (ROC) analysis in the GEO database.

**Results:**

In this study, we identified 54 upregulated and 10 downregulated genes that overlapped between normal and VT samples. After performing WGCNA, the magenta module was the module with the strongest negative correlation with the clinical characteristics. From the key module, FECH, GYPA, RPIA and XK were chosen for further validation. We found that these genes were upregulated in VT samples, and high expression levels were related to recurrent VT. Additionally, the four hub genes might be highly correlated with ribosomal and metabolic pathways. The ROC curves suggested a diagnostic value of the four genes for VT.

**Conclusions:**

These results indicated that FECH, GYPA, RPIA and XK could be used as promising biomarkers for the prognosis and prediction of VT.

**Supplementary Information:**

The online version contains supplementary material available at 10.1186/s12872-021-02409-4.

## Introduction

Venous thromboembolism (VT), which includes pulmonary embolism (PE) and deep vein thrombosis (DVT), has been reported to be the third most commonly occurring cardiovascular disease (CVD) worldwide, following coronary heart disease and hypertension [1]. Annually, approximately 600,000 incidents are diagnosed in the United States. Furthermore, VT leads to complications, such as reappearance, chronic thromboembolic pulmonary hypertension, postthrombotic syndrome, and death [2–6]. Early diagnosis and treatment for VT patients are crucial to effectively reduce mortality and improve prognosis.

In recent decades, considerable attention has been given to exploring new biomarkers and potential molecular mechanisms for VT diagnosis and therapy [7–10]. Based on the GEO database, RPL9, RPL5, RPS20, TP53, and RPL23 were enriched in the ribosome pathway, validating them as potential targets for VT therapy [9]. This is a noteworthy finding, as it suggests that the PAI-1 4G/5G polymorphism might be a prospective VT risk biomarker, especially in the Asian population, according to a meta-analysis [11]. Moreover, it has been reported that COX7C and UQCRQ may play vital roles in a single VT, while ADRBK1, NDUFA5, and ATP5O may be possible targets for recurrent VT [12]. Therefore, it is essential to identify new novel biomarkers significantly correlated with VT diagnosis to improve the effectiveness of therapeutic approaches.

In this research, we examined 2 GEO datasets and found 64 significant differentially expressed genes (DEGs) between normal and VT samples. A weighted gene coexpression network analysis (WGCNA) was performed to evaluate the key module correlated with VT. Moreover, the preservation of gene modules was evaluated as preserved. Four hub genes, FECH, GYPA, RPIA, and XK, were found to be highly associated with VT, including low risk, moderate risk, high risk, single, and recurrent VT. Additionally, four genes appeared to be highly correlated with ribosome and metabolism pathways based on the GSEA and GSVA data. The diagnostic values of the four genes were validated by ROC curves. Thus, we identified the four hub genes FECH, GYPA, RPIA, and XK as new biomarkers and verified the prognostic and predicted values for VT patients.

## Methods

### Collection of data

The RNA expression profiles were obtained from two eligible microarray datasets (GSE19151 [13] and GSE48000 [14]) that contained 107 healthy samples and 160 VT samples. The patients with VT were separated into 3 groups: (1) ‘low-risk’ patients had one or more provoked VTs; (2) ‘moderate-risk’ patients had a single unprovoked VT; and (3) ‘high-risk’ patients had ≥ 2 unprovoked VTs.

## Identification of robust DEGs

The R program “limma” was used to standardize the data and evaluate DEGs based on dataset series matrix files [15]. The DEGs that met the criteria of adjusted *p* value < 0.05 and log 2-fold change (FC) > 0.5 were filtered by robust rank aggregation (RRA) [16].

### Gene ontology (GO) and kyoto encyclopedia of genes and genomes (KEGG) pathway analyses

Using the R package “clusterProfiler”, we conducted GO enrichment analysis for the DEGs; the analysis included molecular function (MF), cellular components (CC), and biological process (BP). KEGG pathway analysis was performed to investigate the high-level functions and utilities of the biological system.

### WGCNA and identification of the key module

We chose the top 25% of genes with the most variance from GEO to build a coexpression network in R using the WGCNA program to identify VT-associated modules. Once outliers were removed at a cutoff point of 10,000, the data were grouped using Pearson’s correlation [17]. The optimum power value was determined once the independence level was 0.9 and a slope of approximately 1 was chosen. Subsequently, for network creation and module identification, we adjusted the soft-threshold power to 4, the cutoff height to 0.25, and the least module size to 10. Gene significance was used to quantify the associations between individual genes and traits.

### Module preservation analysis

The module preservation function (nPermutations = 200) was used to generate module preservation and quality metrics to assess the stability of the obtained module utilizing the WGCNA package [18]. GSE19151 was a validation dataset that comprised mRNA expression data from 133 specimens. The modules showing elevated Zsummary and reduced medianRank scores were considered highly conservative and stable modules, respectively [19].

### Gene set enrichment analysis (GSEA) and gene set variation analysis (GSVA)

We used the R package “clusterprofiler” to conduct GSEA on hub genes using sequencing data [20]. Furthermore, the “GSVA” R program was utilized to identify the pathways most closely associated with hub genes [21]. The samples were categorized into two cohorts based on the median expression per hub gene (low expression vs. high expression). *p* < 0.01 was considered statistically significant. The chosen reference gene set was “c2.cp.kegg.v6.2.symbols.gmt”, which was acquired from the Molecular Signature Database (MSigDB).

### Validation of the prediction of hub genes

We used the “pROC” R package to construct receiver operating characteristic (ROC) curves and compute the area under the ROC curve (AUC) to assess the projected values of hub genes [22].

## Results

### Differentially expressed mRNAs related to VT

**Fig. 1:**
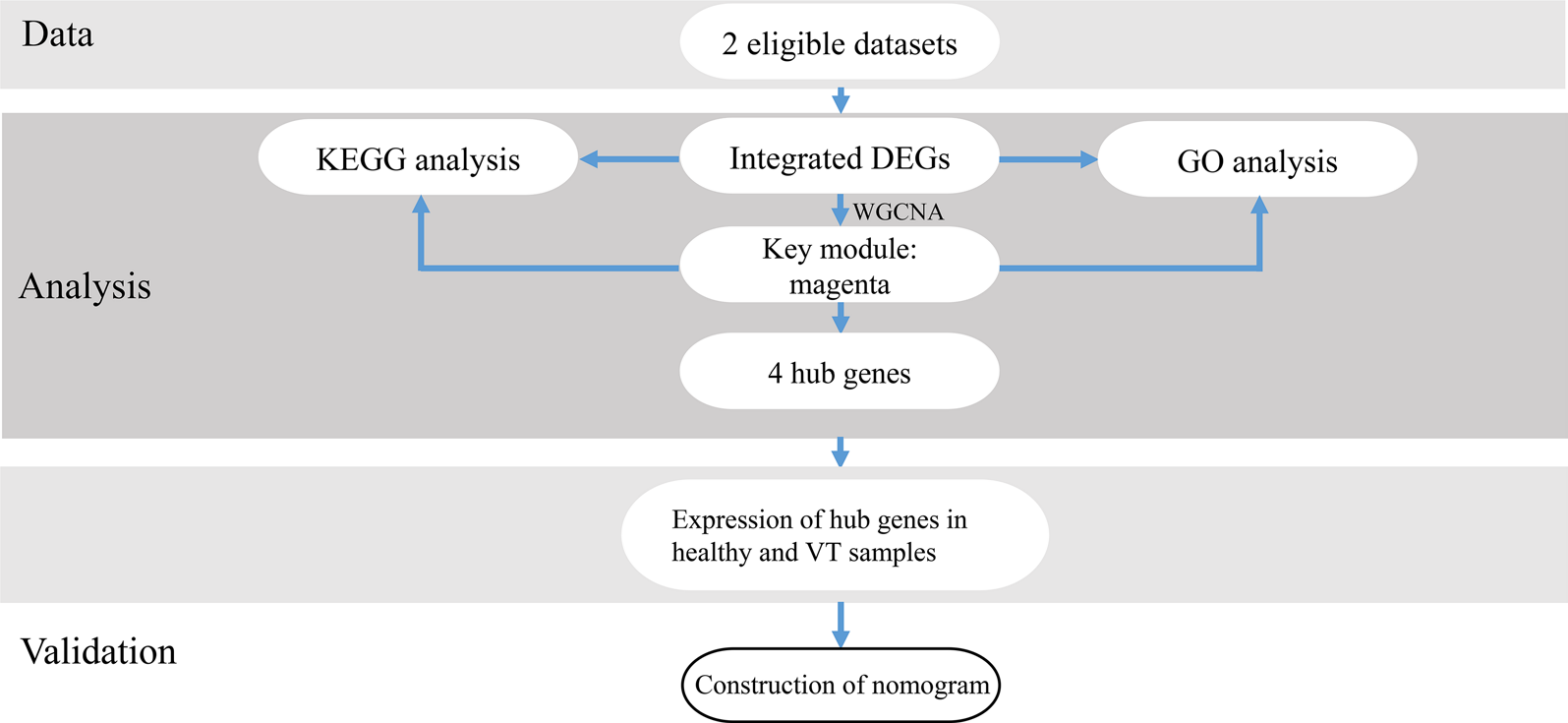
The workflow of this study

**Fig. 2:**
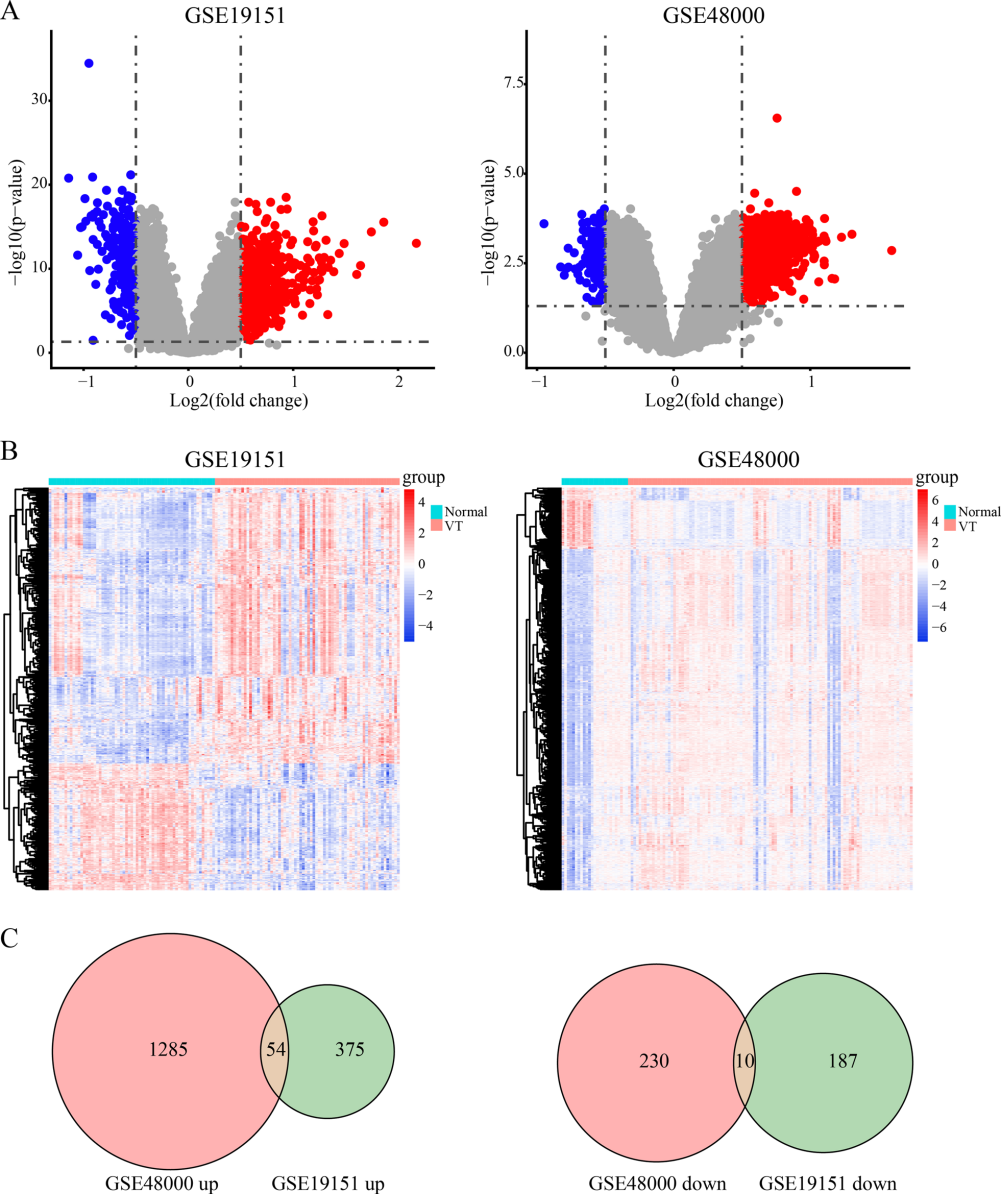
Analysis of DEGs in VT. **a** Volcano plot visualizing DEGs between normal and VT samples in GSE19151 and GSE48000; **b** the expression of DEGs in the heatmap of normal and VT samples; Venn diagrams showing the identification of **c** overlapped upregulated and **d** downregulated DEGs in two different datasets

We compared the gene expression between normal and VT samples by analyzing GSE19151 and GSE48000 (Fig. 1). There were 375 upregulated and 187 downregulated genes in GSE19151 and 1285 upregulated and 230 downregulated genes in GSE48000 (Fig. 2a). The DEGs are presented in the Heatmaps (Fig. 2b). The Venn diagrams show the 54 upregulated and 10 downregulated genes that overlap the two different GEO datasets (Fig. 2c, Additional file 1).

### Pathway enrichment analysis of DEGs

**Fig. 3:**
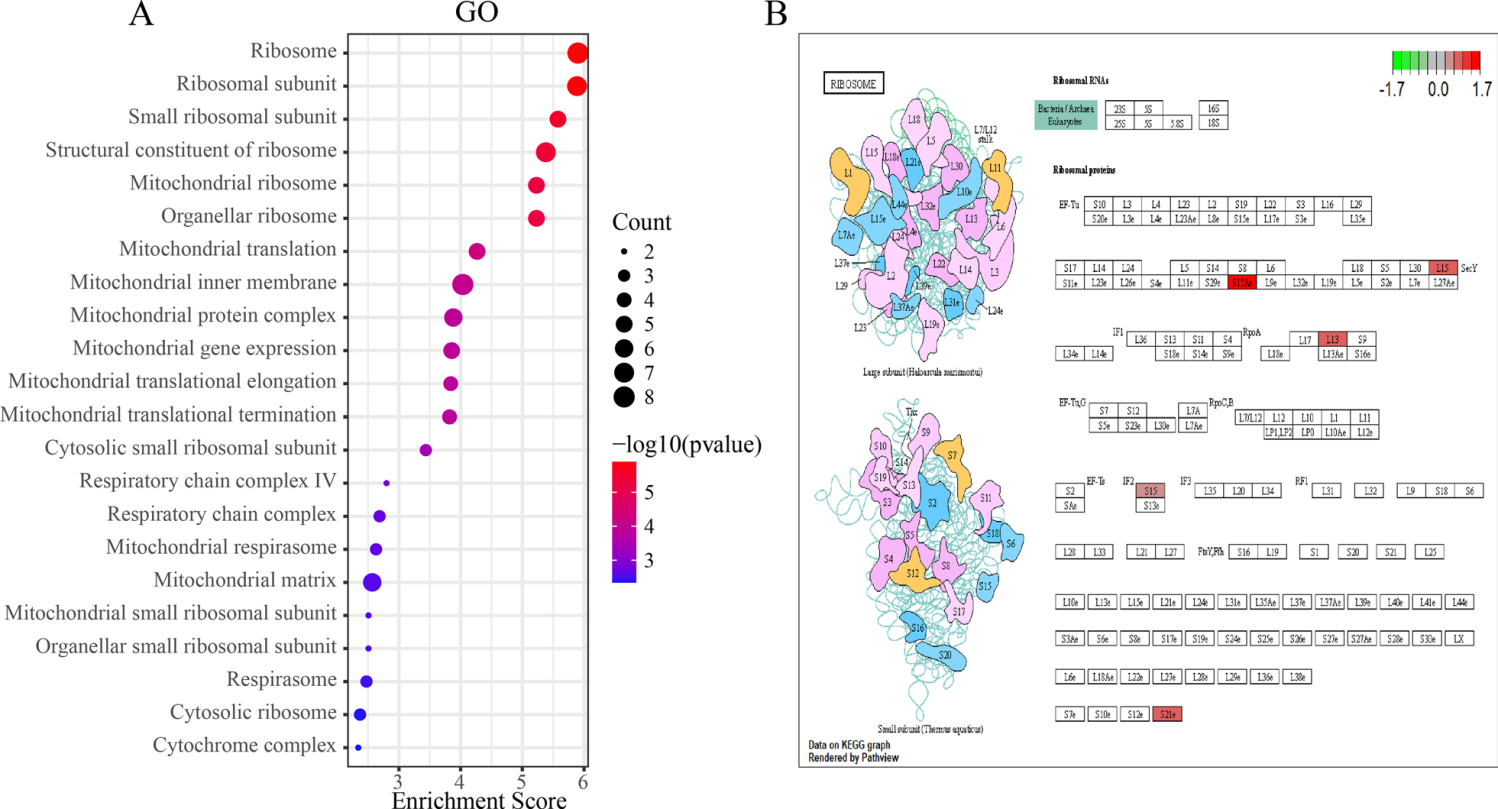
Functional enrichment of DEGs. **a** GO terms identified in the GO analysis for DEGs; **b** Visualization of the ribosome pathway. Red nodes represent upregulated DEGs

The top five enriched pathways for DEGs were ribosomal, structural constituent of ribosome, small ribosomal subunit, ribosomal subunit, and mitochondrial ribosome pathways (Fig. 3a, Additional file 2). In addition, the metabolic pathways were visualized in schemes depicting the ribosomal pathway (Fig. 3b). In the ribosomal pathway, RPS15, RPL15, RPL13, and RPS21 were upregulated. These data reveal that these genes might be crucial for VT.

### WGCNA

**Fig. 4:**
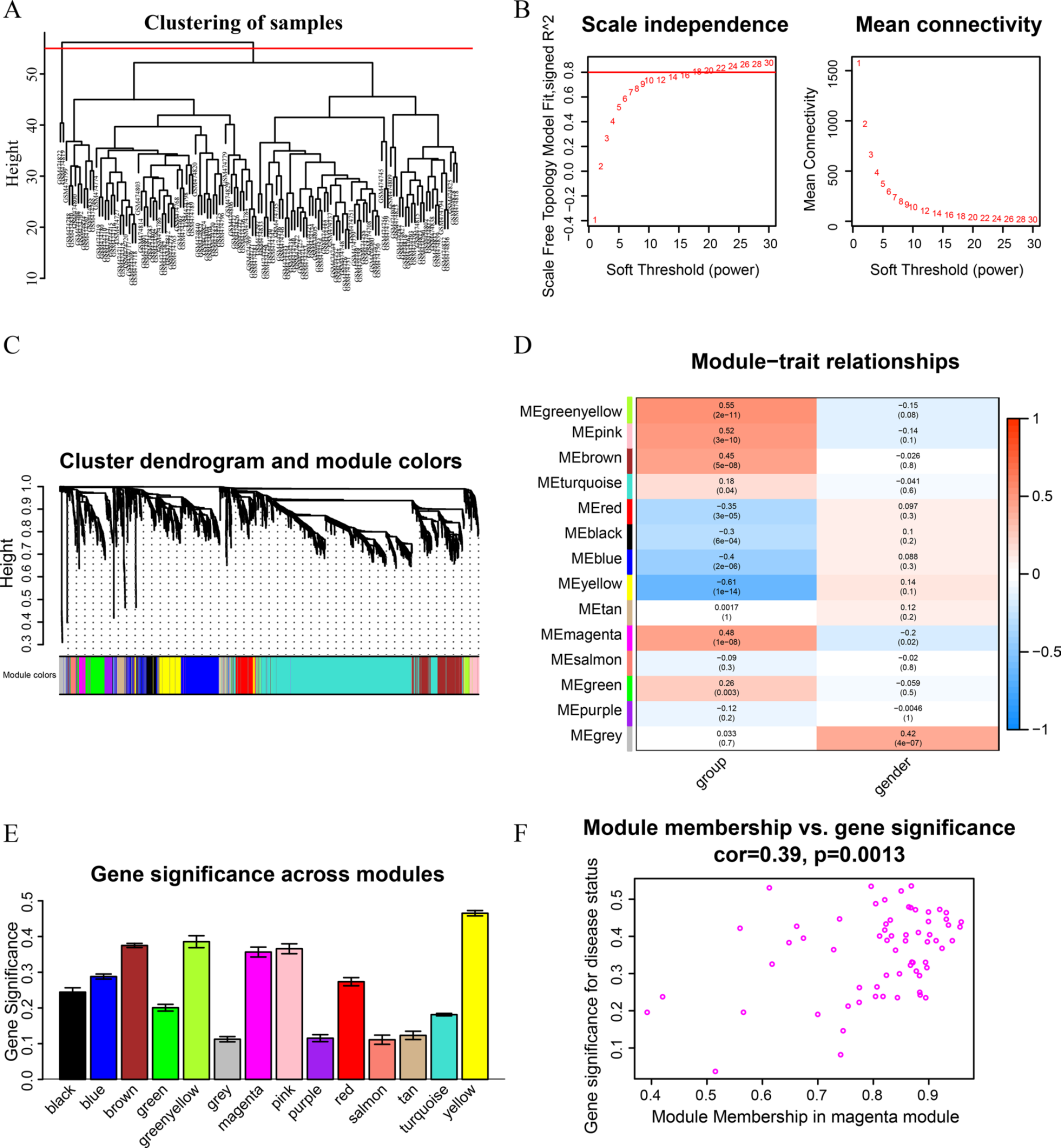
Identification of key modules associated with clinical traits by WGCNA. **a** Clustering dendrograms of samples; **b** Analysis of the scale-free fit index and the mean connectivity for various soft-thresholding powers; **c** Dendrogram of all DEGs clustered with dissimilarity measure based on topological overlap; **d** Heatmap of the correlation between module eigengenes and clinical traits. Each row denoted a module eigengene, each column represented a clinical trait and each cell contained the correlation coefficient and *p* value; **e** Gene significance in different modules (bottom); **f** Scatter plot of genes in yellow module

A WGCNA was performed on GSE19151, integrating the DEGs generated from the RRA analysis to identify the main modules that highly correlate with the clinical characteristics of VTs (Fig. 4a). As indicated in Fig. 4b and c, beta (β) = 4 (scale-free R2 = 0.8) was additionally adjusted as the soft threshold for computation of adjacencies. Moreover, an aggregate of 14 modules was discovered after merging similar modules (Fig. 4c). Based on a heatmap of module–trait associations, the yellow, green–yellow, pink, and magenta modules were the four with the strongest association with VT (Fig. 4d). Additionally, we found that the significance of the yellow, green–yellow, pink, and magenta modules was higher than that of the others module, implying that these modules might have a significant relationship with VT (Fig. 4e). Furthermore, in the magenta module, the association and *p* value between module affiliation and gene significance values were 0.39 and 0.0013, respectively (Fig. 4f).

### Network preservation analysis

**Fig. 5:**
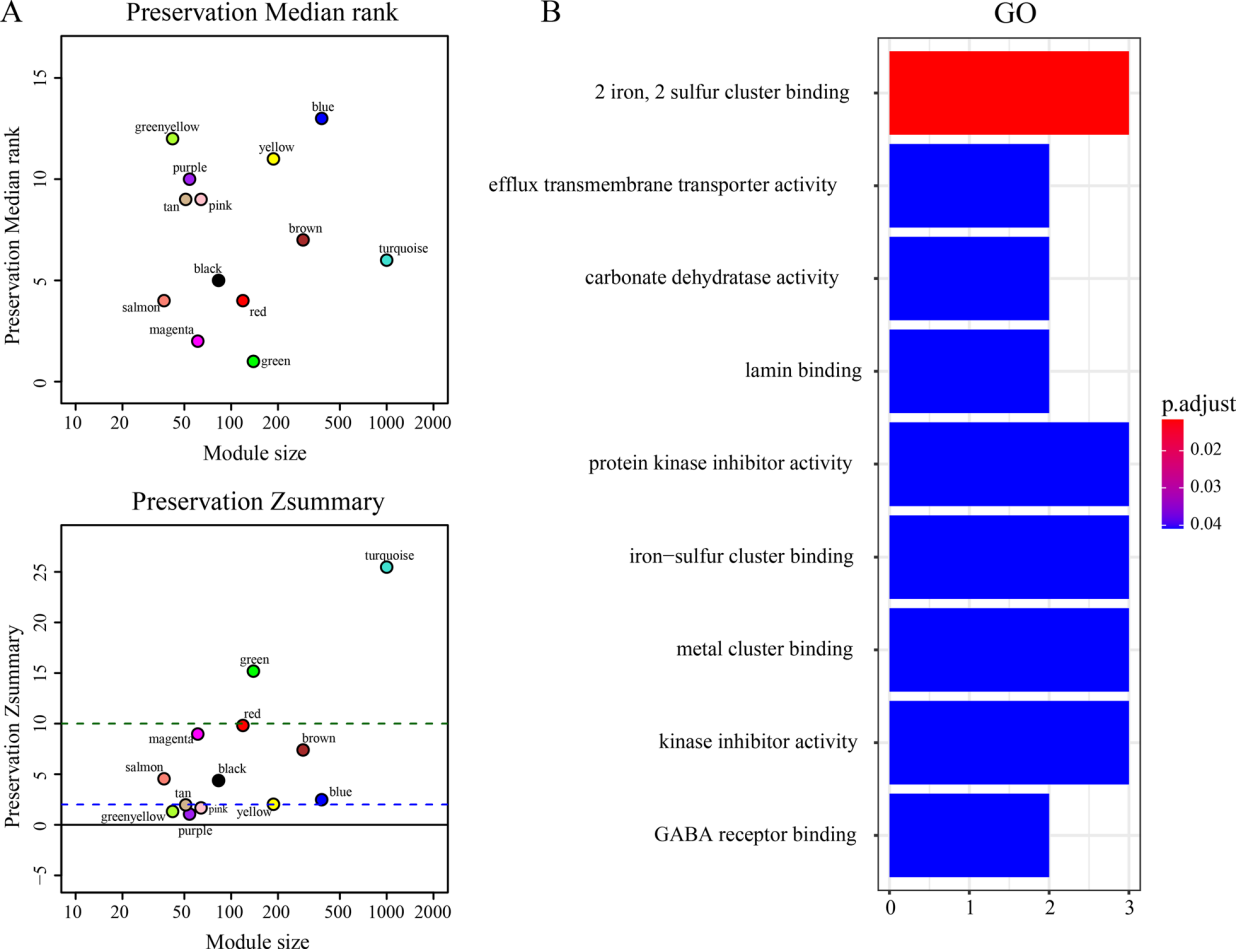
The medianRank and Zsummary values of the module preservation using the GSE19151 dataset. **a** The medianRank graph and the Zsummary graph. The dashed blue and green lines indicate the thresholds Zsummary = 2 and Zsummary = 10, respectively; **b** The plot for the top 9 GO enrichment annotations of all genes in magenta module

We utilized GSE19151 as a validation set to execute preservation analysis to assess the preservation of gene modules. The preservation was evaluated using an integrated modulePreservation algorithm in the WGCNA package. A Z score ranging between 2 and 10 was considered to represent mild to moderate preservation, while scores above 10 represent excellent preservation [23–26]. Meanwhile, a module with a substantially lower rank appears to have better observable preservation metrics than a module with a greater median rank. Combining the median rank, Z score, and module–trait correlations, the magenta module, comprising 65 DEGs, was found to be the module with the strongest negative correlation with clinical characteristics (Fig. 5a, Additional file 3). The most enriched pathways of the DEGs in the magenta module were 2 iron, 2 sulfur cluster binding, efflux transmembrane transporter activity, and carbonate dehydratase activity (Fig. 5b, Additional file 4).

### Validation of hub genes

**Fig. 6:**
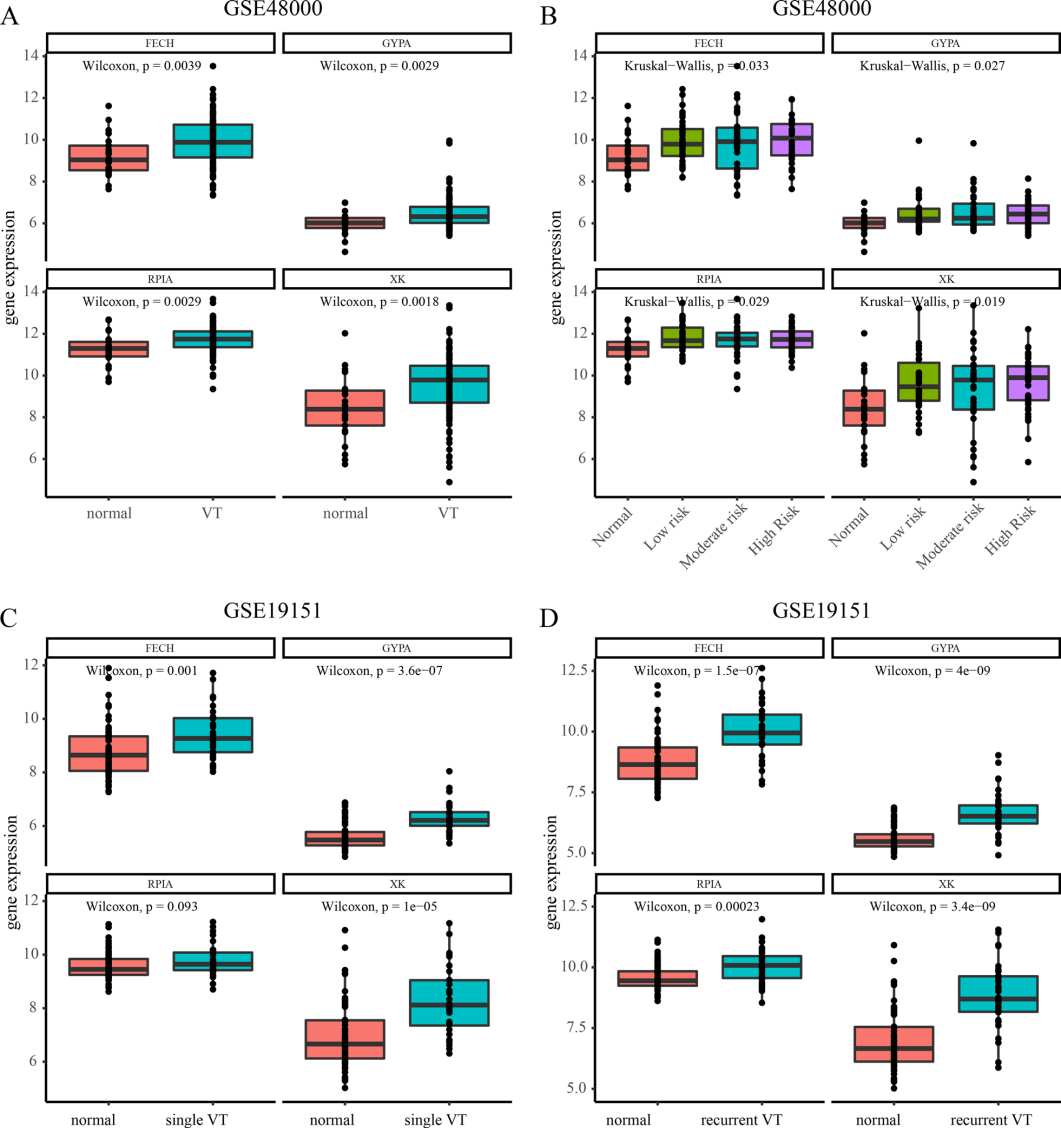
Correlation analysis between the expression of EFCH, GYPA, RPIA and XK and clinicopathological parameters in GSE19151 and GSE48000. **a** the expression of hub genes and VT; **b** low risk and high risk; **c** single VT; **d** recurrent VT

We chose four hub genes (FECH, GYPA, RPIA, and XK) to explore their correlations with clinical values. They were shown to be more highly expressed in the VT group than in the normal group (Fig. 6a). There was a significant difference in the low-, moderate- and high-risk groups compared to the normal group (Fig. 6b). Based on the data from GSE19151, we found that the levels of hub genes were much higher in the single VT (Fig. 6c) and recurrent VT (Fig. 6d) groups than in the normal groups. The data suggested that four genes were strongly correlated with VT and might be associated with VT recurrence.

### GSEA and GSVA of four hub genes

**Fig. 7:**
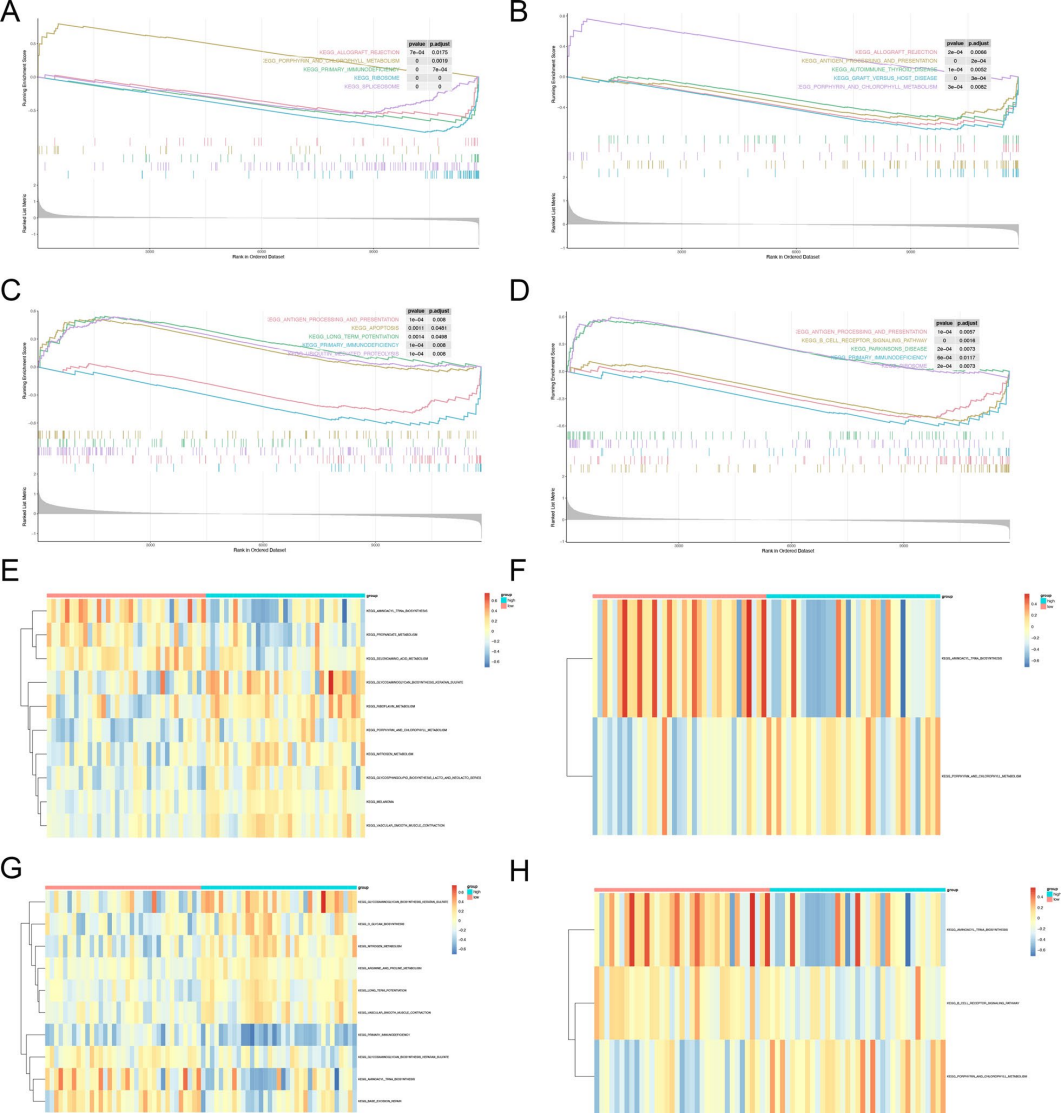
GSEA and GSVA of hub genes in datasets. **a**–**d** Top 3 gene sets (according to GSEA enrichment score) enriched in the high-expression group of **a** EFCH; **b** GYPA; **c** RPIA; **d** XK. GSVA-derived clustering heatmaps of differentially expressed pathways for **e** EFCH; **f** GYPA; **g** RPIA; **h** XK

To further identify the possible functions of FECH, GYPA, RPIA, and XK in VT, we conducted GSEA and GSVA with GSE19151. Genes in the high-expression cohorts of FECH, GYPA, RPIA, and XK were highly enriched in ribosome (Fig. 7a), graft versus host disease (Fig. 7b), primary immunodeficiency (Fig. 7c), and B cell receptor signaling pathways, respectively (Fig. 7d). Based on the analysis of GSVA, FECH was associated with vascular smooth muscle contraction (Fig. 7e). GYPA was enriched in aminoacyl tRNA biosynthesis (Fig. 7f). PRIA was related to nitrogen metabolism (Fig. 7g), and XK was associated with porphyrin and chlorophyll metabolism (Fig. 7h). After comprehensively considering the results of GSEA and GSVA, we concluded that these four genes might be highly correlated with ribosomal and metabolic pathways.

### The predicted values of hub genes in VT

**Fig. 8:**
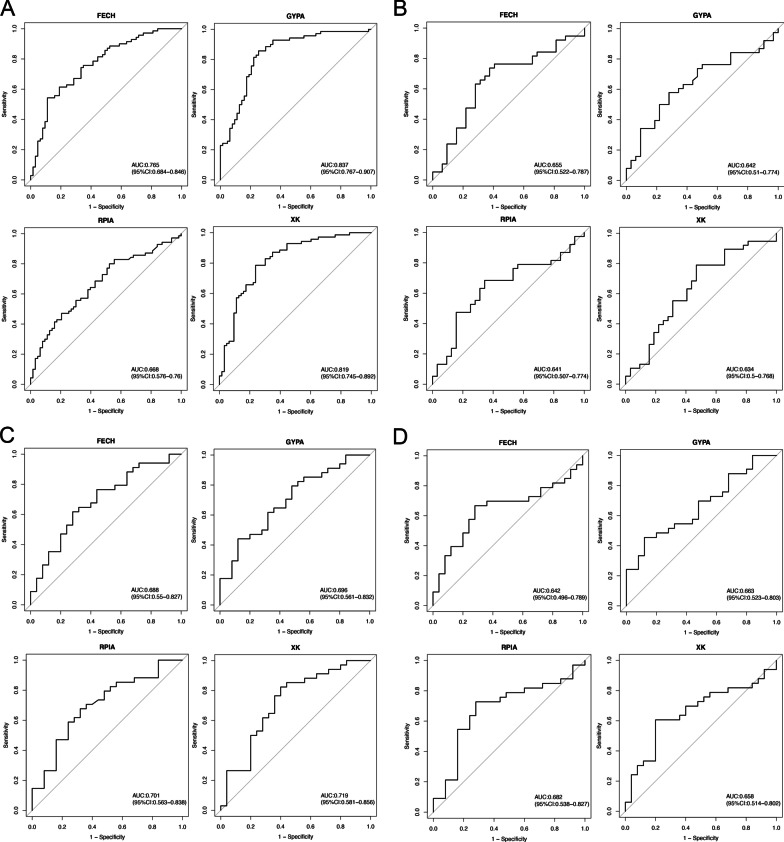
The diagnostic values of hub genes in VT. ROC curves and AUC statistics to evaluate the diagnostic efficiency of hub genes on the incidence of VT (**a**); the possibility of single and recurrent VT (**b**); low risk (**c**) and moderate risk (**d**)

ROC curves showed the predicted value of these genes as biomarkers for the incidence of VT (FECH AUC: 0.765, GYPA AUG: 0.837, RPIA AUG: 0.668, and XK AUG: 0.819) and the possibility of single and recurrent VT (FECH AUC: 0.655, GYPA AUG: 0.642, RPIA AUG: 0.641, and XK AUG: 0.634) (Fig. 8a, b). The ROC curves suggest that these four genes have predictive values for low risk VT (FECH AUC: 0.688, GYPA AUG: 0.696, RPIA AUG: 0.701, and XK AUG: 0.719) (Fig. 8c) and moderate risk VT (FECH AUC: 0.642, GYPA AUG: 0.663, RPIA AUG: 0.682, and XK AUG: 0.658) (Fig. 8d).

## Discussion

VT is reported to be the third most common CVD worldwide following coronary heart disease and hypertension. Although numerous investigations have been conducted to investigate treatment targets for VT, discrepancies between the DEGs discovered have been observed in various studies [27–31]. This study is the first to integrate RRA and WGCNA to identify new biomarkers associated with VT. Two GEO datasets were included in our study, and 54 upregulated and 10 downregulated genes were determined to overlap. After investigating their enrichment in GO and KEGG pathways, we discovered that these DEGs may be involved in ribosomal and metabolic pathways. We were able to identify the coexpression module linked to clinical characteristics using WGCNA. Genes in this module were enriched in 2 iron, 2 sulfur cluster binding, efflux transmembrane transporter activity, and carbonate dehydratase activity.

We identified four hub genes, FECH, GYPA, RPIA, and XK, from the magenta module. High expression of these four genes was significantly associated with VT, low-, moderate- and high-risk VT, and recurrence of VT, indicating the prognostic value of these hub genes. Ferrochelatase (FECH) is the terminal enzyme in heme biosynthesis and plays vital roles in choroidal neovascularization, retinal neovascularization, and erythropoietic protoporphyria [32–34]. It was reported that FECH was involved in the metabolic pathway [35], and our work concurs with this. Glycophorins (GYPA) are one of the primary sialoglycoproteins of the human erythrocyte membrane and serve as receptors for pathogens, including Plasmodium falciparum erythrocyte-binding antigen 175 (EBA-175), influenza virus, and hepatitis A virus (HAV). GYPA plays a vital role in the high activity of solute carrier family 4 member 1 (SLC4A1) and translocation of SLC4A1 to the plasma membrane [36]. Our study showed that GYPA was strongly associated with the occurrence of VT, acting as a possible and not well-studied biomarker for VT. Furthermore, the enzyme ribose 5-phosphate isomerase A (RPIA) plays an essential role in carbohydrate metabolism [37, 38] in Enterococcus faecalis and is a prognostic biomarker for human hepatocellular carcinoma [39]. Our data indicated that RPIA was involved in the incidence of VT. The X-linked Kx blood group (XK) protein has eukaryotic and prokaryotic membrane transport protein structural features. Mutations in XK are highly correlated with McLeod syndrome with defects in the hematopoietic and neuromuscular systems. Thus, FECH, GYPA, RPIA, and XK were identified in our study as potential biomarkers for VT.

To explore their potential biological functions in VT, we conducted GSEA and GSVA, which revealed that these four novel genes might be positively involved in ribosomal and metabolic pathways. It has been reported that RPIA is a vital mediator in the process of carbohydrate metabolism and nucleotide metabolism in cancer [37, 38, 40]. Additionally, many related pathways, such as the B cell receptor signaling pathway, antigen processing and presentation, and allograft rejection, were found to be enriched in the high-expression cohorts of hub genes, revealing their potential roles in VT.

## Conclusions

By combining RRA and WGCNA, we found some robust DEGs and significant gene modules in VT. Four hub genes (FECH, GYPA, RPIA, and XK) were significantly upregulated in the VT cohort, and GSEA and GSVA indicated that these genes might contribute to the incidence of VT. Moreover, these four hub genes were found to be diagnostic biomarkers for the prediction of VT. Further experiments are required to determine the possible mechanisms of these biomarkers in VT.

## Supplementary Information

**Additional file 1**. DEGs of GSE19151 and GSE48000.

**Additional file 2**. GO terms of DEGs.

**Additional file 3**. 65 DEGs in the magenta module.

**Additional file 4**. The enriched pathways of the DEGs in the magenta module.

## Data Availability

The gene expression profiles of GSE19151 and GSE48000 were downloaded from Gene Expression Omnibus (GEO).

## Abbreviations

VT: Venous thromboembolism
RRA: Robust rank aggregation
DEGs: Differentially expressed genes
WGCNA: Weighted gene co-expression network analysis
GSVA: Gene set enrichment analysis
GSEA: Gene set variation analysis
PE: Pulmonary embolism
DVT: Deep vein thrombosis
CVD: Cardiovascular disease
GO: Gene ontology (GO)
KEGG: Kyoto encyclopedia of genes and genomes
MF: Molecular function
CC: Cellular components
BP: Biological process
ROC: Construct receiver operating characteristic
AUC: Area under the ROC curve
FECH: Ferrochelatase
GYPA: Glycophorins
RPIA: Ribose 5-phosphate isomerase A
XK: X-linked Kx blood group
EBA-175: Erythrocyte-binding antigen 175
HAV: Influenza virus, and hepatitis A virus
SLC4A1: Solute carrier family 4 member 1
